# Child Nutritional Status in the Changing Socioeconomic Region of the Northern Amazon, Brazil

**DOI:** 10.3390/ijerph15010015

**Published:** 2017-12-23

**Authors:** Mônica P. L. Cunha, Rejane C. Marques, José G. Dórea

**Affiliations:** 1 Fundação Universidade Federal de Rondônia, Porto Velho CEP 76801-059, RO, Brasil; 2 Department of Nutrition, Faculty of Health Sciences, Universidade de Brasilia, Brasília CEP 70919-970, DF, Brasil; jg.dorea@gmail.com; 3 Universidade Federal do Rio de Janeiro, Campus Macaé, Rio de Janeiro CEP 27930-560, RJ, Brasil; rejanecmarques@globo.com

**Keywords:** nutritional status, child, anthropometry, socio-environmental

## Abstract

The living conditions (i.e., socioeconomic, healthcare-related, nutritional, and environmental) to which children are exposed may influence their ability to reach their optimal growth potential. This review focuses on the relationship between the nutritional status of children under five years of age and social and environmental factors in Northern Brazil. Children living in this region have limited access to healthcare and face precarious socioeconomic and environmental conditions. This analysis was based on data from national health surveys, the consolidated food, nutrition surveillance system (SISVAN), and indicators of the DPSEEA (driving force, pressure, state, exposures, health effects, and actions) framework. The northern region has the worst living conditions in the country, and children under five years of age have significant height-for-age, weight-for-age, and weight-for-height deficits. Concomitantly, the prevalence of children who are overweight has increased significantly, although it remains lower than that in more developed Brazilian regions. Insufficient and/or inadequate dietary practices and early exposure to unfavorable living conditions are risk factors for nutritional deviations. Further advances in public health policies that consider regional characteristics, particularly in the north, where progress has been slower, are needed.

## 1. Introduction

Growth is an essential component in the evaluation of children’s health. Children’s physical development is also an important indicator of the general well-being of a society, because it reflects not only socioeconomic and food safety conditions but also environmental conditions [1]. The living conditions (i.e., socioeconomic, healthcare-related, nutritional, and environmental) to which children are exposed during the intrauterine period and childhood may influence their ability to reach their maximum growth potential [2].

According to an analysis of the external influences on the growth process in Brazilian children, existing regional inequalities are closely linked to differences in the socioeconomic development among regions and investments in sanitation and health care. In Brazil, due to differences in the standard of living among regions and even within the same region, national public policies have focused on inequities in the health conditions in certain localities and vulnerable groups. However, efforts to resolve these regional inequalities are slow and irregular [3].

For example, in the northern region, the national development policy for road construction, colonization projects, and hydroelectric dams has contributed to economic growth, infrastructure, and socio-demographic changes. This policy has resulted in a direct impact on the environment [4,5] and, consequently, on human health by leading to changes in living conditions and the incidence of endemic infectious and parasitic diseases [4]. The northern region encompasses 45% of the territory of Brazil. However, this region has the lowest population density due to the Amazon rainforest, which hinders human habitation. Nevertheless, this region has the largest proportion of young people (0–19 years of age), who account for 40% of the total population [6]. The children living in this region have limited access to healthcare and face precarious socioeconomic and environmental conditions; overall, adequate housing, a clean water supply and waste disposal are lacking or insufficient. This narrative review focuses on the relationship between the nutritional status of children under five years of age and environmental factors in Northern Brazil, which is an area that contains most of the Amazon rainforest.

## 2. Methods

To consolidate information regarding children’s health in this rapidly changing region in Northern Brazil, we reviewed studies and national surveys investigating the nutritional status of children under five years of age. This is a narrative review addressing (1) height and weight deficits, (2) anthropometric indices and environmental influences, (3) overweight and (4) feeding practices and children’s growth. Due to the scarcity of published information in scientific journals, available data were obtained from the National System of Health Information. This analysis was based on data from the following national health surveys: National Study of Household Expenditures [*Estudo Nacional de Despesas Familiares*] (ENDEF)/1974-75 [7]; National Survey on Health and Nutrition [*Pesquisa Nacional de Saúde e Nutrição*] (PNS)/1989 [8]; two Demography and Health [*Demografia e Saúde*] surveys (PNDS)/1996 [9] and 2006 [10]; and the Nutrition Review of the Northern Region [CNRN-*Chamada Nutricional da Região Norte*]/2007 [11]. Nutritional information (including breastfeeding) was available from the Breastfeeding Research Survey [*II Pesquisa de Prevalência do Aleitamento Materno*] (2008) [12] and the Family Budget Surveys [*Pesquisa de Orçamentos Familiares*] (POF)/2002 [13] and 2009 [14].

The ENDEF [7], PNS [8], PNDS [9], and POF [13,14] surveys followed a national protocol based on a probabilistic sampling model targeting socioeconomic strata and clusters; the CNRN [11] and the Breastfeeding Research Survey [12] were conducted during the one-day countrywide pediatric immunization campaigns. Due to the challenges of obtaining up-to-date information on height and weight from national surveys, other resources, such as the consolidated Food and Nutrition Surveillance System [*Sistema de Vigilância Alimentar e Nutricional*] (SISVAN) report in 2015, were used. The SISVAN data are available on the website of the Department of Primary Care known as SISVAN/Web. The SISVAN is an ongoing monitoring system of nutritional status run by the government in the Brazilian Health System (including the National Family Health Program). Monthly reports capture the nutritional status of children according to the Ministry of Health protocol [15]. Socio-environmental factors were selected based on the indicators of the DPSEEA (driving force, pressure, state, exposures, health effects, and actions) framework because these factors indicate the existence of risk conditions or situations that may influence children’s health [16]. 

## 3. Results and Discussion

### 3.1. Height and Weight Deficits

The anthropometric height-for-age (H/A) deficit, which is also known as stunting, is the most representative child growth impairment measure and is the most common form of malnutrition in developing countries [17]. In Brazil, using the first national survey conducted in 1974/75 as a reference, the height deficit decreased significantly from 32.9% to 6.8% by 2006 [3].

Economic growth in Brazil has reduced certain socioeconomic inequalities, particularly in terms of improvements in maternal schooling, family income, maternal and child health care, water supply, and basic sanitation. The improvements in 2007 and 2008 significantly decreased the likelihood of malnutrition among children from poorer families [3,18].

**Figure 1:**
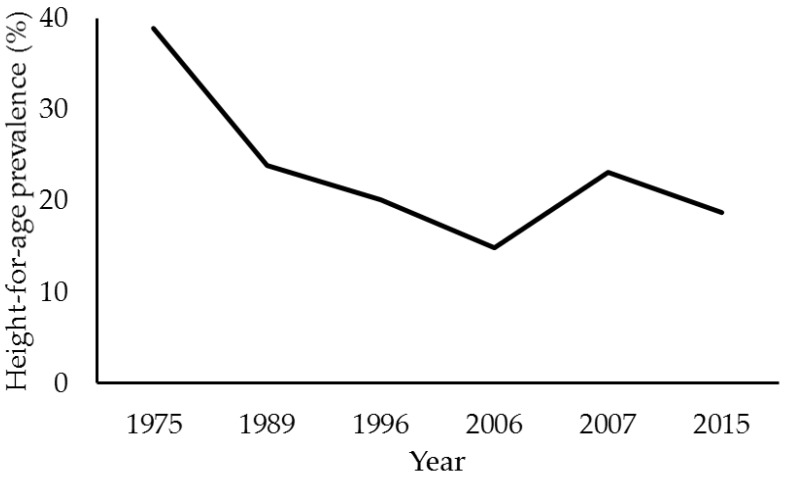
Evolution of height-for-age prevalence (%) in children in Northern Brazil from 1975–2015. Source: National surveys of anthropometry and food consumption [7,8,9,10,11] and SISVAN/Web [15]

Although the growth trajectory showed a marked decline in the prevalence of height deficits, the socioeconomic benefits were not equally distributed throughout Brazil [3,18]. In 2007, the prevalence of H/A deficits reported by the Nutrition Review of the Northern Region was three times higher than the national average reported by the National Demographic and Health Survey in 2006 [10] (Figure 1). These prevalence rates were even greater in the Amazonian states of Amapá (31%) and Amazonas (29.5%) [11].

These regional disparities have also been observed in other regions worldwide. In Ghana, according to a demographic survey, preschool children exhibited a reduction in H/A deficits (from 28% to 19%) over a six-year period; however, in certain regions, the prevalence rates remained high (33%) [19].

While the consolidated data from the SISVAN/2015 showed a lower percentage than that in 2007, the prevalence of H/A deficiency in children remained high (18.7%) [15] (Figure 1). Data from the national surveys indicate that among the Brazilian regions, the northern region has the highest prevalence of H/A deficits, which is greater than the northeastern region, an area known for a high prevalence of height deficits [3].

The H/A deficits in children in the northern region were more prevalent among males (25.3%) than among females (21%) [11]. Although these sex differences remain poorly understood, data from 16 demographic health surveys conducted in 10 sub-Saharan African countries revealed that boys were more prone to impaired height than girls. Thus, a low socioeconomic status is likely related to the higher vulnerability of this group to unfavorable environmental conditions and a higher risk of morbidity and mortality in early childhood [20].

In general, children in developing countries have a greater tendency to sustain height deficits during the most vulnerable period, which is up to two years of age [1,21]. In the northern region, the height deficit during this critical period was demonstrated by CNRN/2007 among children between 12 and 23 months of age (28.2%) [11] and, according to SISVAN/2015, between six months and two years of age (23.3%) [15]. During this stage, the early identification of growth delays is fundamental to prevent stunting from becoming irreversible and from resulting in negative long-term consequences [1,21].

Similar to the national H/A deficits, the weight-for-age (W/A) deficits were higher in 2007 (5.2%) than those reported by PNDS/2006 (3.4%), and the state of Amazonas had the highest percentage (9.7%). According to the POF/2008-09, the northern region was also one of the Brazilian regions with the highest rate of W/A deficits (8.5%) [14].

Globally, the proportion of children with W/A deficits declined from 25% to 14% between 1990 and 2015; however, in 2015, approximately 95 million underweight children under five years of age resided in less-developed regions [22]. In Brazil, according to the consolidated data from SISVAN/2015, the northern region, which is one of the poorest regions in the country, had the highest percentage of W/A deficits (6.1%), surpassing the reported national average of 3.9% [15] or, according to PNDS/2006 [9] and the CNRN/2007 [10], of 3.4% and 5.2%, respectively. In the same survey period, the W/A deficit in the north region was higher than that of Latin America and the Caribbean (3%), Central America (3.7%), South America (2.7%), Western (4.3%) and Central Asia (3.5%), and Northern Africa (5.5%) but less than that of Oceania (18.4%) Southern Asia (28.8%), Western Africa (19.4%), and Africa (15.9%) [23].

Although discrepancies in the anthropometric indicators exist between the national surveys and the SISVAN, a positive trend toward weight deficit reductions in the north region was observed between 1974 (24.5%) and 2015 (6.1%).

The weight-for-height (W/H) deficit is another indicator of the nutritional status; acute cases of malnutrition are commonly identified in the second year of life [1,21], and acute malnutrition is related to infectious diseases, inadequate feeding practices, and food insecurity. W/H deficits tend to constitute reversible challenges [21]. However, the increased frequency of W/H deficits in children may indicate a greater risk of delayed linear growth [21,24,25].

Although W/H and H/A are measurements that provide important information about the overall health of children and help predict child growth and developmental disorders, these measures are independent and are presented individually [24,25].

In the northern region, the PNDS/2006 (0.7%) [10] and SISVAN/2015 (5.8%) results [15] differ using the W/H deficit as an indicator of nutritional status. The W/H deficits in 2015 are more than five-fold greater than those in 2006, which is certainly due to the derivation of the SISVAN data from a larger percentage of children who had greater access to the Basic Health Units of the National Health System and who may have been more exposed to determinants of poor nutritional status.

Compared to large population surveys, monitoring systems, such as SISVAN, provide information regarding the nutritional status over time more rapidly and at a lower cost. However, these data should be analyzed with more attention, particularly those provided by SISVAN, because those data include children who are a part of the public health system, which may comprise populations that are more vulnerable to nutritional disorders. 

### 3.2. Anthropometric Indices and Environmental Influences

The relationship between children’s growth and environmental factors has been used to explain the different growth patterns among populations. According to a multicenter study conducted in eight different locations, the genetic characteristics of each population had only a slight influence on the height variation in the children; notably, in 80% of the cases, the differences in height were related to environmental factors [2].

In Latin America, the influence of environmental factors on children’s health is related to known risks, such as water contamination, emerging factors related to climate change and exposure to toxic contaminants [26]. Children under five years of age who live in unfavorable socioeconomic conditions in low- and middle-income countries have greater exposure to poverty, malnutrition, poor housing conditions, and poor basic sanitation, which directly influence their healthy development [27]. Due to the socioeconomic heterogeneity in Brazil, an unfavorable environment is expected to influence child growth. The northern region of Brazil has the highest percentage of people (24%) living in households located in subnormal clusters (favelas and similar), which is almost twice the national average (12.5%) [16]. This region also has the second highest proportion of people living below the poverty level (earning less than ¼ of the minimum wage), which is surpassed only by the northeast region [6].

**Figure 2:**
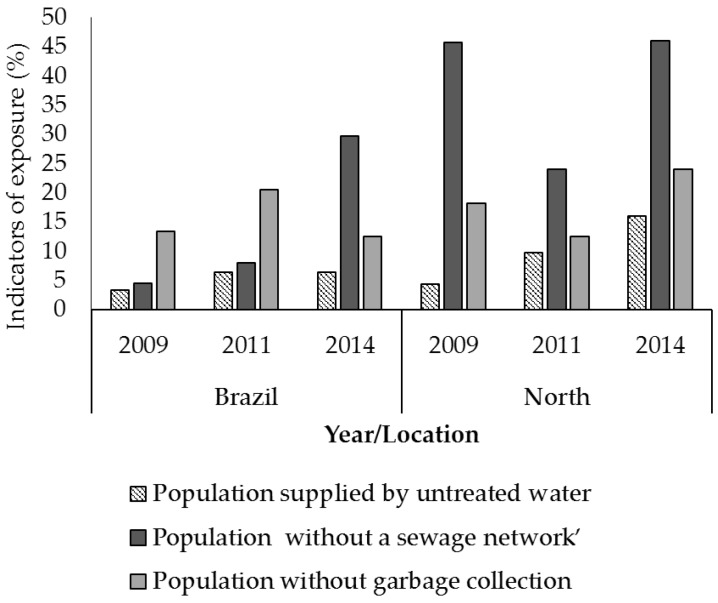
Indicators of exposure (DPSEEA framework) in Brazil and the northern region. Source: National Health Surveillance Agency, 2014 [16].

Another negative factor influencing children’s growth is the lack of basic sanitation (i.e., a clean water supply, sanitary sewage systems, and garbage collection). Due to the natural environment in the Amazon rainforest, the northern region has the worst sanitation conditions (Figure 2), resulting in the highest percentage nationwide of hospitalizations due to diseases related to inadequate environmental sanitation [15].

In 2015, according to the Brazilian National Health Survey, poor basic sanitation was observed in 9.6% of homes with children up to 14 years of age; in the northern region, this percentage reached 19.2% [6]. The lack of access to basic sanitation services is consistent with the occurrence of diarrhea in this region. Furthermore, the hospitalization rate for acute diarrhea in children under five years of age is the highest in the country (15.74%) [16].

In addition to poor sanitation, Amazonian children are exposed to other environmental challenges, such as high levels of air pollution caused by intentional and unintentional fires. The northern region has the highest occurrence of fires in Brazil, and fires have been shown to affect human health and birth weight [28]. Moreover, air pollution exposes children to a greater risk of respiratory complications; children are more vulnerable to the hazardous effects of gaseous pollutants and particulate matter than adults [29].

In children under five years of age, air pollutants, environmental changes (e.g., forest burns), low socioeconomic status, and malnutrition are determinants of increased cases and aggravation of respiratory infections [29,30,31]. In the northern region, hospitalization rates for acute respiratory infections in children less than five years of age are higher than those of any other Brazilian region, including large urban centers [16].

In addition to air pollution, children living in North Brazil, where rapid urbanization, poor housing, and lack of sanitation coupled with intensive mining and agricultural projects (compromising natural resources and traditional living) are occurring are predisposed to a greater probability of exposure to pollution and chemical contamination [4,5]. In the northern region, 10,415,597 people (approximately 32% of the total population in Brazil) are estimated to live in areas contaminated or suspected of being contaminated [16] with mining wastes (toxic metals) and industrial and urban residues [32].

Major hydroelectric ventures in this region have had significant socio-environmental effects, such as their impact on population explosion, resettlement, loss of livelihoods, insect proliferation, declining living conditions (health, education, transport, and basic sanitation), increased greenhouse gas emissions, deforestation, reduction in the quantity of fish species, and mercury methylation, which is the most toxic form for humans [4].

The determinants of growth in children include a complex set of variables involving socioeconomic status and unsanitary environments. Indeed, children’s growth reflects not only the well-being of children but also the existing health and nutritional inequalities among populations [27,33].

### 3.3. Overweight

Childhood overweight and obesity are risk factors for an increased incidence of noncommunicable diseases in adults, such as hypertension, type 2 diabetes, cardiovascular diseases, endocrine disorders, certain types of cancer, and sleep apnea, [34] among others. In addition to the increased susceptibility to chronic diseases, overweight and obesity also predispose individuals to negative economic and social consequences in adulthood [1,35].

**Figure 3:**
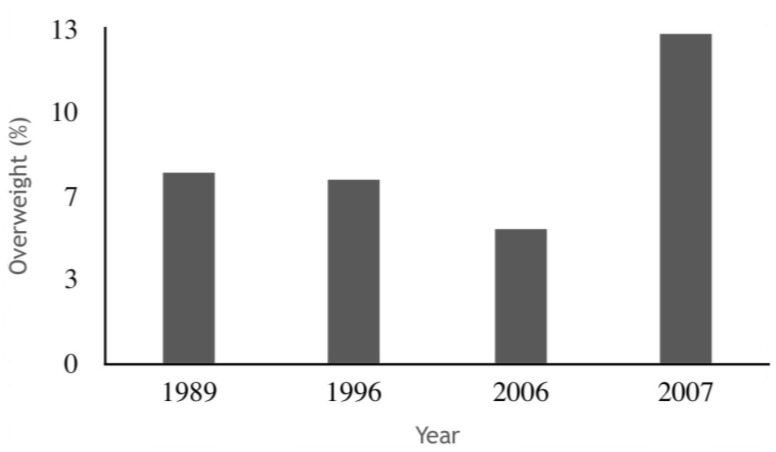
Prevalence of overweight children under five years of age in the north region from 1989–2007. Source: National anthropometry and food consumption surveys [8,9,10,11]

Concomitantly with the occurrence of the anthropometric deficits, childhood malnutrition may manifest as excess weight. Between 1990 and 2015, the prevalence of overweight children worldwide, including in developing countries, increased from 4.9% to 6% [36]. In Brazil, anthropometric data from the ENDEF of 1974-75 and the POF/2008-09 showed that the increments in the weight measurements were higher than the increments in the height measurements [14]. In the northern region, the prevalence of overweight for height reported by the CNRN/2007 (12.8%) [11] exceeded that presented by PNDS/2006 (5.2%) [10] (Figure 3). Additionally, the CNRN data showed that children of African or indigenous descent and those residing in rural areas were at increased risk for becoming overweight [11].

Overweight problems in early childhood may be influenced by race/ethnicity and socioeconomic conditions [37,38]. Using a detailed ethnic classification of two cohorts (United Kingdom and United States) involving approximately 30,000 children, Zilanawala et al. [38] observed that those from ethnic minorities had a greater risk of becoming overweight. The disparities between whites and other racial/ethnic groups in the UK and USA demonstrated that in these groups, health and socioeconomic disadvantages may be a risk factor for child overweight [38]. 

In poor regions, such as Northern Brazil, the prevalence of overweight and obesity in children has increased. In 2015, this prevalence was 12.9% [15]. Notably, according to the nutritional surveys, the northern region had the greatest deficit in stature, which indicated that obesity is not only prevalent among the most socioeconomically favored but also may be a risk factor for children belonging to low-income families with a history of prior malnutrition [39].

Due to increased processes of nutritional transition in low- and middle-income countries, a new nutritional profile has been reported in children under five years of age in which the height-for-age deficit and excess weight occur simultaneously, and the lifelong effects resulting from this condition are unknown [40].

During the early years of life, the basis for the body composition in adulthood is formed, and fat cell growth occurs more rapidly during this period [34]. Therefore, the period from conception to two years of age is considered critical for the development of childhood obesity and its adverse consequences [34,41,42]. Indeed, the CNRN/2007 found the highest percentages of children with excess weight [11]. During this stage, several prenatal (higher maternal body mass index (BMI) and smoking) and infant (high birth weight and rapid weight gain) factors have been consistently associated with late childhood obesity [35].

Although overweight and childhood obesity are currently a focus of public health worldwide due to their association with obesity in adulthood and other adverse consequences, studies in Brazil on the prevalence of overweight and obesity in children and adolescents in different Brazilian regions are limited, and evidence needed for public policies is lacking [42].

### 3.4. Feeding Practices and Children’s Growth

The nutritional transition currently experienced in Brazil has been marked by malnutrition (under- and overweight) [43,44], which is a known risk factor for acute and chronic diseases [1]. Balanced nutrition, particularly during the period from conception to the second year of life, has a beneficial health effect in adulthood. Large-scale nutritional interventions, such as the promotion of breastfeeding (BF), have shown favorable short- and long-term results [45,46,47].

The numerous benefits derived from BF were confirmed by Victora et al. [45]. These authors claimed that 823,000 deaths in children under five years of age could be avoided annually if BF practices were universalized. Additionally, BF might contribute to a reduction in sudden infant deaths, respiratory infections, and diarrhea; lower the risks for developing obesity and diabetes; and provide long-term beneficial effects on intelligence. The health and developmental benefits of BF may result in lower expenditures among health systems worldwide [45].

Due to the importance of BF in all populations regardless of the socioeconomic level, several of the following initiatives were implemented in Brazil: expansion of human milk banks, trade limitations of breast milk substitutes, and increased maternity leave. These BF promotion policies have led to significantly improved breastfeeding in Brazil over the previous three decades, serving as a reference for other countries [45,47].

According to the Breastfeeding Prevalence Survey II (PPAM-2008), compared to other Brazilian regions, northerners performed better in BF practices during the first year of life. Nevertheless, an early introduction of water, teas, and other milks during the first month of life was reported [12]. However, children of six to 12 months of age consumed high amounts of unhealthy foods, such as soda and coffee, and solid food before the sixth month of life. Children older than two years frequently consumed artificial juices, jellies, soda, snacks, and filled biscuits. Another factor considered unfavorable to the healthy growth and development of children was the consumption of sugar and its by-products [11,12].

The age at which solid food is introduced, the quality of the diet, and the dietary habits of the family are important for achieving optimal nutritional targets [48]. Together, these factors may constitute determinants of long-term health. Early weaning and complementary feeding predispose children to obesity and other comorbidities [49]. Since infant feeding is influenced by the family, we might infer that the food consumption of the Brazilian population—among both adults and children—is marked by a diet low in nutrients with a high-energy value and differentiated profiles of food availability among the macro-regions [43].

In the northern region, milk-based preparations, including porridges, are heavily consumed [11]. The high intake of milk contrasts with the low intake of good nutritional quality foods, such as beans, fruits, and vegetables [13,14,43]. However, other foods, such as cassava flour and fish, are preferred by those living in the north region of the country [14,50]. The intake of fish by northerners is approximately 95.0 g/day [14]. In 2013, the proportion of people over 18 years of age who consumed fish at least once per week was 77.2% [43].

In general, Brazilians have a low average consumption of fish (17.3 kg/year), which approximates the global per capita consumption (18 kg/year) [51]. Unlike other regions in the country, due to the abundance of rivers, fish diversity, and socioeconomic and cultural factors, the Amazonian population has a higher preference for fish [50,52,53]. Amazonians consume an average of 369 g/person/day of fish, or 135 kg/year [51]; in certain traditional riverside populations in the Madeira River, the average daily fish consumption reaches 406 g/day and 148.2 kg/year [52].

Amazonian fish are recognized as an important protein resource, which helps balance a high consumption of starchy foods [50,53]. Additionally, fish contain omega-3 fatty acids, which are beneficial for fetal and child development [54]. However, advisories to pregnant and breastfeeding women exist to avoid consuming fish with high Hg content. In a study conducted by Stratakis et al. (2016), in which they followed 26,184 pregnant women and their children, the authors found that eating fish more than three times per week during pregnancy was associated with an increased risk of developing childhood obesity [55].

Exposure to endocrine disrupters during the critical period of development might be associated with changes in the functioning of the endocrine system that contribute to the development of obesity [56]. However, factors such as the lack of a biomarker of fatty acids and insufficient information regarding environmental pollutants in fish, the specific species consumed and their origin are limitations noted by the study [52].

Caution is warranted in generalizing this association, particularly to populations with a traditional lifestyle in the Amazon, which are characterized by high fish consumption, prolonged breastfeeding and exposure to several contaminants in fish. In these populations, in contrast to European and American populations, the weekly fish consumption, even when correlated with the presence of mercury in children, does not affect birth weight [57] or linear growth in breastfed children [58].

Maintaining a balanced diet in childhood is important for optimal growth and development. However, although Amazonian fish are good sources of selenium and nutrients [50,53], the population in the north region consumes a low-quality diet due to a high intake of energy-protein, cholesterol, and polyunsaturated fatty acids and highly prevalent inadequate nutrient consumption, such as that of iron and folate [13,14,43]. 

Bortolini et al. (2015) evaluated the dietary practices of children aged six to 36 months in Brazil and concluded that those residing in the north were more highly vulnerable because they had a lower chance of having a high-quality, diversified diet [44]. The socioeconomic inequalities experienced in childhood may determine lifelong health and eating patterns. Exposure to inadequate nutrition, particularly in children under five years of age, results in health risks in adulthood.

Recognizing that the influence of the environment along with nutritional factors on growth may overlap with genetically determined factors, it is possible to infer that nutritional deviations in children in the northern region tend to reflect early exposure to unfavorable living conditions because the anthropometric markers reported here are important predictors of the future.

### 3.5. Limitations

In the transitioning socioeconomic environment of the vast Amazon territory, migration, agricultural projects, mining (clandestine and legal) and hydroelectric projects have attracted migrant workers and families and have disrupted the traditional living of these populations. The impact of these factors on the nutritional status of children cannot be ascertained with the used methodologies; environmental issues (migration and attending changes in living conditions) are not addressed in these anthropometric surveys, thus limiting our understanding of the determining factors on children health. 

Besides this, secondary data is another limitation to be considered in regards to the distinct methodologies used and the time of the surveys; some of them are ‘official government reports’, with all their inherent limitations (inadequate sample size, irregular timing, and issues related data collection and aggregation). The individual methodologies of the national health surveys were originally designed for specific purposes and may have worked well for their objectives. It is clear that we still need regional surveys that consider sociocultural differences between urban, rural, and subsistence-based communities. These living settings in the Amazon region are quite distinct from any of the regions found in Brazil. Additionally, the SISVAN data collection system reflects a standard protocol requiring specialized and well-trained personnel not available in remote places of the Amazon. These difficulties may compromise data quality for the regional peculiarities of the Amazon. Despite such shortcomings, the results emphasize that children under five years of age in the northern region of Brazil have significant deficits in H/A, W/A, and W/H. Therefore, SISVAN remains the only system capable of assessing differences in nutritional status to guide policies to attenuate persisting inequalities in children health. While these are important limitations, their recognition can be useful to guide future research and health policies for the region.

## 4. Conclusions

Understanding ‘environment and health’ is central to promote children’s health and prevent nutrition deficits common in developing economies. This is especially important in transitioning economies that frequently promote economic growth but do not necessarily provide health improvements for traditional populations.

Data extracted from monitoring systems, such as SISVAN, compared to large population surveys, make it possible to provide information on nutritional status over time more quickly and at a lower cost and to assess differences in nutritional status to guide policies to attenuate persisting inequalities in child health. Therefore, the results emphasize that children under five years of age in Brazil’s northern region have significant deficits in H/A, W/A, and W/H. The insufficient and/or inadequate dietary practices in the northern region are disturbing because these factors are risk factors for growth deficits. Furthermore, the prevalence of overweight, although lower than that of more developed Brazilian regions, has significantly increased compared to that of populations living under better socioeconomic conditions. Despite the important socioeconomic changes in urbanization and healthcare that have occurred over the previous decades in Brazil, regional and social inequalities are concentrated in the poorer regions, such as the northern region, which has the worst living conditions in the country (e.g., serious social problems related to poor housing, unsanitary environments, and lack of basic sanitation).

This finding reconfirms that the substantial efforts in Brazil to reduce malnutrition have not progressed equally among the macro-regions. Moreover, the nutritional transition phase experienced by the poorest population indicates the need for interventional actions that integrate health, environment and socioeconomic development issues. These actions are particularly needed in the northern region, where progress in infant health has been slower.
